# Correction: Inequalities in population health loss by multiple deprivation: COVID-19 and pre-pandemic all-cause disability-adjusted life years (DALYs) in Scotland

**DOI:** 10.1186/s12939-022-01714-4

**Published:** 2022-08-25

**Authors:** Grant M. A. Wyper, Eilidh Fletcher, Ian Grant, Oliver Harding, Maria Teresa de Haro Moro, Diane L. Stockton, Gerry McCartney

**Affiliations:** 1 Place and Wellbeing Directorate, Public Health Scotland, Glasgow, Scotland; 2 Data Driven Innovation Directorate, Public Health Scotland, Edinburgh, Scotland; 3 Directorate of Public Health, NHS Forth Valley, Stirling, Scotland; 4 Clinical and Protecting Health Directorate, Public Health Scotland, Edinburgh, Scotland

**Correction to: Int J Equity Health 20, 214 (2021)**

**https://doi.org/10.1186/s12939-021-01547-7**

After publication of this article [1], the authors reported that in the section ‘Results’, in the sentence that reads ‘The RII was 1.16, which when divided by 0.5 and expressed as a percentage means that the rate in the most deprived areas was 58% higher than the mean rate of the population’ the term ‘divided’ should have read ‘multiplied’.

The original article [1] has been corrected.
